# Evaluation of the protective and therapeutic effects of *Pistacia atlantica *gum aqueous extract on cellular and pathological aspects of experimental asthma in Balb/c mice

**Published:** 2019

**Authors:** Zaynab Shakarami, Hadi Esmaeili Gouvrchin Ghaleh, Bahman Mansouri Motlagh, Ali Sheikhian, Bahman Jalali Kondori

**Affiliations:** 1 *Department of Microbiology, Faculty of Veterinary Medicine, Urmia University, Urmia, Iran.*; 2 *Razi Herbal Medicines Research Center, Lorestan University of Medical Sciences, Khorramabad, Iran.*; 3 *Applied Virology Research Center, Baqiyatallah University of Medical Sciences, Tehran, Iran.*; 4 *Faculty of medicine, Baqiyatallah University of medical sciences, Tehran, Iran.*

**Keywords:** Asthma, P. atlantica, BALB/c mouse

## Abstract

**Objective:**

The purpose of this study was to investigate the protective and therapeutic effects of aqueous extract of *P. atlantica *gum on an experimental asthma in BALB/c mice.

**Materials and Methods:**

Aqueous extract of dried and milled *P. atlantica *gum was assemble and evaluate by GC-MS. In order to investigate the effect of *P. atlantica *gum extract on cellular and pathological aspects of asthma, 60 BALB/c mice were divided into six groups as: negative control, asthmatic group, asthmatic group receiving dexamethasone (1mg/kg; intraperitoneal (IP)) and three asthmatic groups receiving different concentrations of the extract (100, 200 and 400 mg/kg, orally) from the beginning of the study and continued for 84 days. The examined parameters included cell population, IgE antibody production, levels of IL-4, IL-5, TGF-β, INF-γ, IL-10, and IL-17 cytokines, and lung tissue damage.

**Results:**

Regardless of the dose, aqueous extract of *P. atlantica *gum, caused significant decrease in the number of BALF eosinophilic cells and levels of anti-ovalbumin IgE, IL-4, IL-5 and IL-17 cytokine levels, as well as pathologic damage of the lung tissue. In addition, the amount of anti-inflammatory IL-10, TGF-β, and INF-γ Th1 cytokines significantly increased in the extract-treated groups compared to the asthmatic and dexamethasone-treated groups. Moreover, IFN-γ/IL-4 ratio significantly increased in a dose-dependent manner compared to the un-treated asthma group.

**Conclusion:**

The aqueous extract of *P. atlantica *gum can be considered as a potent anti-inflammatory and immunomodulatory compound and may be used as a natural compound for treatment of immune system disorders.

## Introduction

Protective and therapeutic effects of medicinal plants such as *Cynodon dactylon*, *Sphaeranthus indicus *Kurz, *Striga orobanchioides *Benth, *Mucuna pruriens*, *Momordica dioica*, *Piper betel *Linn, and *Olea europaea *were proved in asthmatic patients (Taur et al., 2011 ▶). Although synthetic drugs have considerable efficacy, they often have many adverse side effects (Ashokkumar et al., 2013 ▶). The wild pistachio (Baneh), *Pistacia atlantica*, attached to the Anacardiaceae family, which is often grown in the eastern, western and central parts of Iran (Minaiyan et al., 2015 ▶). Researchers have reported the presence of active compounds such as saturated fatty acids (e.g. palmitic acid), unsaturated fatty acids (e.g. palmitoleic acid), terpenoids, oleic acid, linoleic acid, tocopherols, tocotrienols and phenolic compounds such as gallic acid, in the plant extract. Various studies showed that these compounds possess antioxidant and anti-inflammatory properties. In traditional medicine, *P. atlantica *gum extract is used to treat hepatic diseases, digestive disorders, neurological problems, and parasitic infections, and is employed for treatment of *Helicobacter pylori* infection and respiratory problems (Katzung et al., 2015 ▶; Sharifi et al., 2011 ▶; Taran et al., 2010 ▶). 

Allergic asthma is a chronic inflammatory disorder of the respiratory system, which is associated with increased leukocyte infiltration, especially eosinophils in the lung tissue, increased mucosal secretion, and impaired respiratory function (Ober et al., 2011 ▶). Chronic inflammatory disorder of the respiratory system causes polarization of the immune system responses to T helper 2 (Th2) and increases the production of interleukin-4 (IL-4), IL-5 and IL13 cytokines. These cytokines alter the isotype/class of antibodies to IgE and increase eosinophil infiltration into the respiratory tract (Tsai et al., 2013). One of the most important goals considered in the research for the treatment of allergic respiratory diseases and asthma, is the specific suppression of inflammatory mediators to change Th2 responses to Th1 by increasing the secretion of interferon gamma (INF-γ), IL-12 and tumor necrosis factor-β (TNF-β). 

Regulatory T (T reg) cells are a subdivision of T lymphocytes that play an important role in lung homeostasis and induce the tolerance of immune cells to harmful antigens. Several mechanisms have been proposed for the suppressive effects of regulatory T-cell cells on effector cells including production of repressive cytokines such as tumor growth factor-β (TGF-β), IL-10 and IL-35, inducing metabolic disturbances in target cells, and ultimately functional suppression of dendritic cells. T helper 17 (Th17) lymphocytes play a key role in inflammatory lung disease. This cell produces IL-17 cytokine that stimulates the smooth muscle of the lung tissue (Newcomb et al., 2013 ▶). 

In herbal medicine, therefore, the main goal is to use compounds that alter immune responses from Th2 and Th17 to Th1 type and induce the regulatory T cells. This study evaluated the immunomodulatory and protective and therapeutic effects of aqueous extract of *P. atlantica *gum on the cellular and pathological aspects of an experimental asthma model in BALB/c mice.

## Materials and Methods

***P. atlantica*** **gum extraction**

Fresh *P. atlantica *gum was purchased from Khorramabad grocery stores, Lorestan, Iran. A herbarium collection manager at the University of Khorramabad identified and specified the gum (Herbarium No. HLu27061397). After rinsing/drying, the gum was powdered and vacuum-dried using an electric mill. The resulting powder was then soaked in distilled water for 48hrs before filtration. The extract was incubated at 50°C, and then subjected to re-distillation (Hosseini et al., 2013 ▶).

**GC-MS analysis**

For GC-MS analysis was performed by a Shimadzu GC-17A (Kyoto, Japan) gas chromatograph coupled to a Shimadzu Quadruple-MS model QP5050 mass spectrometer. Compounds were separated on a 30m×0.22mm i.d. fused-silica capillary column coated with 0.25µm film of BP-5 (Shimadzu) and a split/splitless injector with an internal glass liner of 1mm. Ultra-pure helium was used as a carrier gas and the ionization voltage was 70eV. The injector and interface temperatures were 280 and 260^◦^C, respectively. Mass ranged from 35 to 450amu. The oven temperature range was the same as that mentioned above for the GC. The extract constituents were identified by calculation of their retention indices under temperature-programmed conditions for n-alkanes (C8–C20), and for the oil on a DB 5 column under the same chromatographic conditions. Genotype compounds were identified through comparison of their mass spectra with those of the internal reference mass spectra library (NIST08 and Wiley 9.0) (Abtahi Froushani et al., 2016 ▶).

**Asthma induction by ovalbumin (OVA)**

Ovalbumin (OVA) is a frequently used allergen derived from chicken egg, which induces a robust (Nails et al., 2008 ▶). OVA (4μg) was dissolved in 1000μl of PBS buffer and mixed with an equal volume of Al (OH)_3_ gel. Then, 100μl of the solution was injected intraperitoneally (IP) to animals by G27 needle on days 0, 7, 14 and 21. From days 27 to 31, they were sensitized with OVA aerosol (20 ng OVA in 50µl PBS) for 30 min, three times a week for 12 weeks (i.e. 84 days). The mice were first anesthetized (ketamine 50 g/kg/Xylazine 10mg/kg) and then, 150 μl of a solution containing 50 μg/μl of OVA in sterile PBS, was poured into their nostrils. Control group treated in the same way with PBS without OVA (Lee et al., 2008 ▶).

**Study population and test groups**

**Figure 1:**
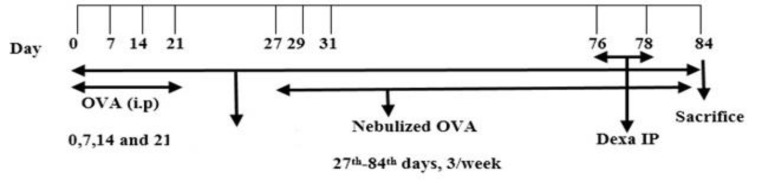
The Schematic timeline of the study

The most commonly used strain of mouse for antigen challenge models is BALB/c as it develops a good Th2-biased immunological response (Nails et al., 2008 ▶). Therefore, the study population included 60 male BALB/c mice aged 6 to 8 weeks, purchased from the Pasteur Institute of Iran. Animals were kept for two weeks before the beginning of the study. In order to conduct experiments, animals were randomly divided into six groups of 10 mice (Table 1). Asthma was induced by OVA in five groups, and one group of healthy mice was considered control group. Three asthmatic groups concurrently received the aqueous extract of *P. atlantica *gum orally (100, 200 and 400mg/kg per day-orally) from the beginning of the study and continued for 84 days and one asthmatic group received dexamethasone (1mg/kg; IP) on the 76^th^ and 78^th^ days of the study. Also, a group of asthmatic group received normal saline until the end of the study. The study lasted for 84 days as schematically shown in Figure 1. The research performed under the supervision of the ethics committee of the research on laboratory animals, the University of Khorramabad, Lorestan, Iran. Animal care and the general protocols used for animal management were done in compliance with the regulations of the Ministry of Health and Medical Education of I. R. of Iran, approved by the Medical Ethics Committee of the Lorestan University of medical Sciences (IR.LUMS.REC.1397.165).

**Table 1:** Treatment protocol used for different groups of animals

Group	Abbreviation	characteristics
Control	C	PBS (Orally)
Asthmatic	A	PBS (Orally)
*P. atalantica Gum *Extract 100	E-100	PAGE (Orally-100 mg/kg)
*P. atalantica Gum *Extract 200	E-200	PAGE (Orally-200 mg/kg)
*P. atalantica Gum *Extract 400	E-400	PAGE (Orally-400 mg/kg)
Dexamethasone	Dexa	Dexa (IP-1 mg/kg)

**Measurement of specific OVA IgE and preparation of bronchoalveolar lavage fluid (BALF):**

At the end of the challenge, the heparinized blood samples were collected by cardiac puncture under Ether anesthesia. Serum samples were isolated by centrifugation and IgE production was determined using an ELISA kit (Cayman, USA) according to the manufacturer’s instructions. Simultaneously, ice-cold PBS (0.5 mL) was instilled twice into the whole lung tissue, followed by aspiration of BALF. After centrifugation (at 1200 rpm for 10 min at 4°C), cells were counted (at least 200 cells per slide) using a hemocytometer (Chuanfeng et al., 2015 ▶; Zhenghao et al., 2016 ▶).

**Assessment of cytokines in culture supernatants**

Splenocytes were aseptically isolated from mice, and cell suspensions (2×10^6^ cells/ml) were cultured in 24-well plates and stimulated with 50μl OVA solution (200µg/ml) as described by Abtahi et al. (2015) ▶. The supernatant was removed after 72hrs and cytokines production was determined using an ELISA kit (Peprotech, Iran). The ratio of INF-γ/IL-4 was calculated after converting the unit of interferon gamma (INF-γ, ng/ml) to pg/ml. Cytokine ELISA kit worked based on the standard principle of a sandwich enzyme-linked immunosorbent assay. A 96-well plate was coated with specific mouse monoclonal antibodies for each cytokine. Standards and test samples were added to the wells and cytokines present in the sample are bound by the immobilized antibody. A cytokine-specific biotinylated polyclonal antibody from goat was added subsequently. After washing away the unbound biotinylated antibody with Phosphate-buffered saline (PBS) or Tris Buffered Saline (TBS), avidin-biotin-peroxidase complex was added to the wells. The wells were washed again with PBS buffer to remove the unbound conjugates. Horseradish peroxidase (HRP) substrate TMB was used to visualize the HRP enzymatic reaction. TMB was catalyzed by HRP to produce a blue product that turns into yellow after adding an acidic stop-solution. The density of yellow color is proportional to the cytokine captured onto the plate. The OD (optical density) values were measured by an ELISA reader at 450nm wavelength. The cytokine amount for each sample was calculated using standard curves derived from standard samples (Abtahi Froushani et al., 2014 ▶).

**Histological examination of lung tissue**

For this purpose, the mice were killed by spinal cord injury, and the lung tissue was completely isolated and placed in 10% formalin solution. The lung tissues were dissected from the middle zone of the left lung and fixed in 4% neutral-buffered formalin. Samples were then prepared with Hematoxylin and Eosin staining for histopathological examinations done under a light microscope. The results of staining were scored from 0 to 3 based on the levels of inflammatory cells infiltration, thickening of the muscle layer, and mucus increase. Scores 0, 1, 2, and 3, respectively, indicated that there were no, low, moderate, and severe inflammation. Finally, values obtained for each parameter were collected to express the severity of the lesions (Esmaili Gourvarchin Galeh H., 2018 ▶).

**Statistical analysis**

Data are presented as mean±SD. One-way analysis of variance (ANOVA), followed by Dunnett's test, was performed to uncover significant differences between the parametric data. Non-parametric data (i.e. histopathological scores) were analyzed by Mann-Whitney U test. A p<0.05 was considered statistically significant.

## Results

**GC/MS analysis**

As shown in Table 2, Alpha-pinene and β-pinene comprised 77.9% and 3.66% the aqueous extract of *P. atlantica *gum, respectively.

**Histopathology**

**Figure 2:**
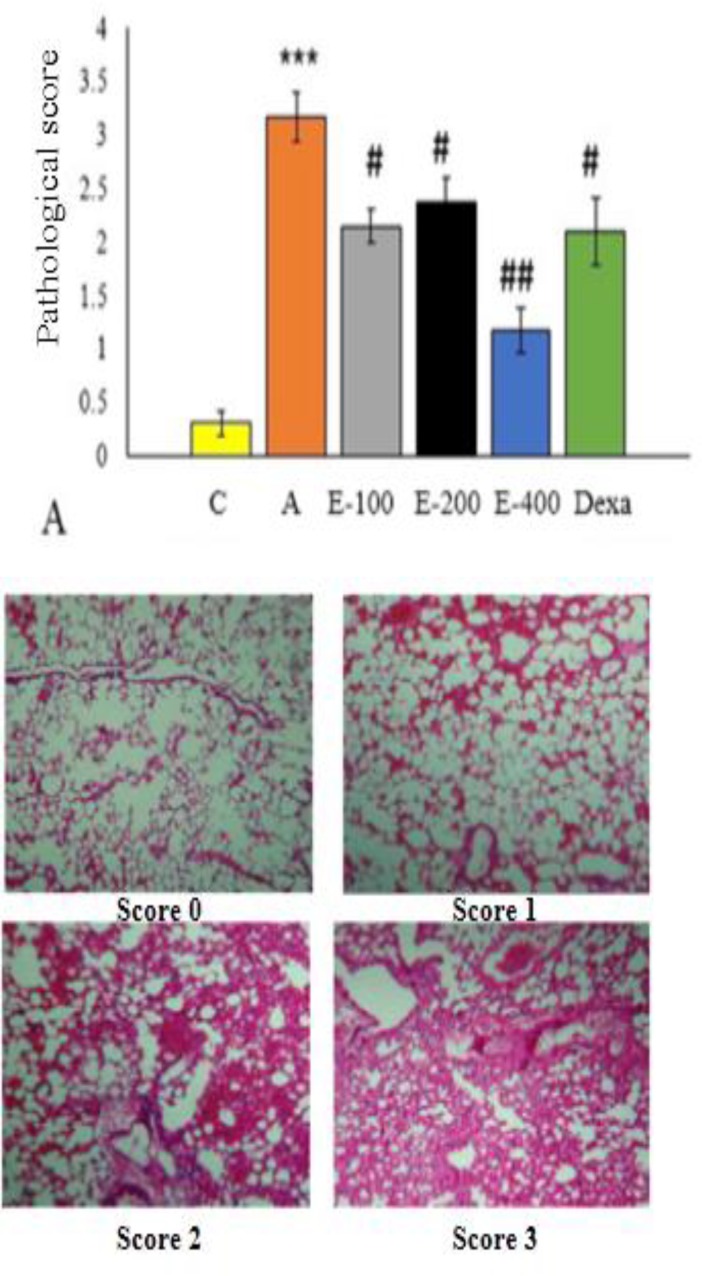
A. Results of blind analysis of histopathological sections of the middle zone of the left lung in different groups. C (control), A (asthmatic), E-100, 200 and 400 (*P. atalantica Gum *extract 100, 200 and 400) and Dexa (dexamethasone)). B. Representative sections depict scoring system used for histopathological examinations (X400). Score 0: no inflammation was observed; Score 1: inflammatory cells were occasionally observed; Score 2: most bronchi were surrounded by a thin layer of inflammatory cells; Score 3: most bronchi or vessels were surrounded by a thick layer of inflammatory cells. ***p<0.001, asthmatic group compared to C group. #p<0.05, ##p<0.01, treated groups compared to asthmatic group

The results of microscopic examination of the slides (Figure 2) showed that tissue damage mostly occurred in the asthmatic group. So, inflammatory cell infiltration, thickening of the muscular layer and mucus accumulation increased in almost all lung tissues (>90%), and the degree of pathological damage was 3 in this group. In the control (C) group, pathological changes were not observed, which is equal to the lowest pathological score (zero). In groups treated with different concentrations of aqueous extract of *P. atlantica* gum (100, 200 and 400mg/kg) and dexamethasone, the number of inflammatory cells, muscle layer thickening, and mucus accumulation were significantly lower than those of the asthmatic groups in which pathological damage score 1 for epithelial destruction was observed (p<0.01) (Figure 2).

**Table 2:** Concentration (%) of different compounds in aqueous extract of *P. atalantica *gum

**Compound**	**RI**	**Amount (%)**
Alpha-Pinene	939	77.9
Camphene	953	0.88
Verbenene	967	0.43
Beta-Pinene	980	3.66
Beta-Myrcene	991	6.26
Delta-3-carene	1011	0.24
Alpha-Terpinene	1018	0.03
P-Cymene	1026	0.3
L-Limonene	1031	0.76
1,8-Cineole	1033	0.14
Trans-beta-Ocimene	1050	0.18
Gamma-Terpinene	1062	0.07
Terpinolene	1088	0.21
Verbenol	1098	0.13
Linalool	1103	0.08
Chrysanthenone	1146	0.09
Rans-Limonene-Oxide	1149	0.59
Pinocarveol	1152	1.7
Cis-Sabinol	1149	1.93
Isopinocamphone	1181	0.14
Pinocarvone	1162	0.05
P-Mentha-1,5-dien-8-ol	1185	1.02
Terpinene-4-ol	1190	0.25
P-Cymen-8-ol	1199	0.55
Myrtenol	1190	0.66
Verbenone	1204	0.24
Cis-Carveol	1241	0.15
Isobornyl acetate	1302	0.2

Additionally, the tissue damage in mice treated with 100 and 200mg/kg of the extract or 1mg/kg of dexamethasone improved similarly (p<0.05). The reduction in lung tissue inflammation in the group treated with 400 mg/kg extract was significantly higher compared to the asthmatic group (p<0.001). It is noteworthy that the protective effects of the extract were not concentration-dependent. 

**Assessment of BALF**

**Figure 3:**
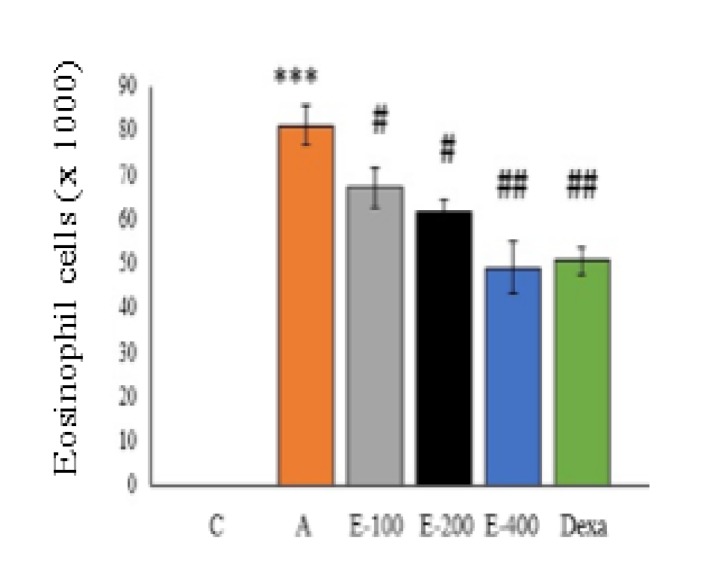
Bronchoalveolar lavage fluid eosinophilic cell count. Asthmatic (A) group compared to control group, ***p<0.001. Different treated groups compared to asthmatic group, #p<0.05, ##p<0.01

**Figure 4:**
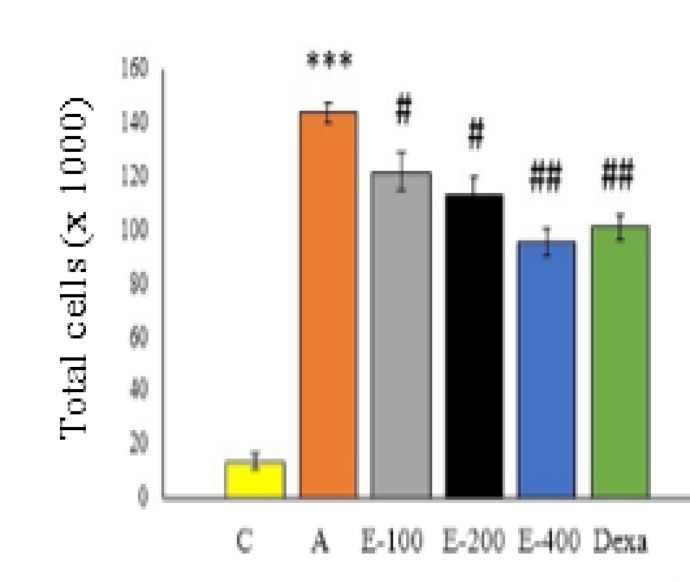
The effect of aqueous extract of *P. atalantica *gum on bronchoalveolar lavage fluid cell count. Asthmatic (A) group compared to control group, ***p<0.001. Different treated groups compared to asthmatic group, #p<0.05, ##p<0.01

As shown in Figures 3 and 4, administration of OVA significantly increased the number of eosinophilic cells and total number of cells in the asthmatic (A) group compared to control (C) group (p<0.001). Oral administration of the aqueous extract of *P. atlantica *gum at all concentrations and dexamethasone significantly reduced the number of eosinophilic cells and total number of BALF cells compared to the asthmatic group (p<0.05 to p<0.01). The number of eosinophils and total number of cells in 400 mg/kg extract treated group were significantly lower compared to asthmatic group (p<0.01). Although a more pronounced decrease was recorded in the concentration of the extract, this reduction was not dependent on the concentration. 

**Assessment of IgE production**

**Figure 5:**
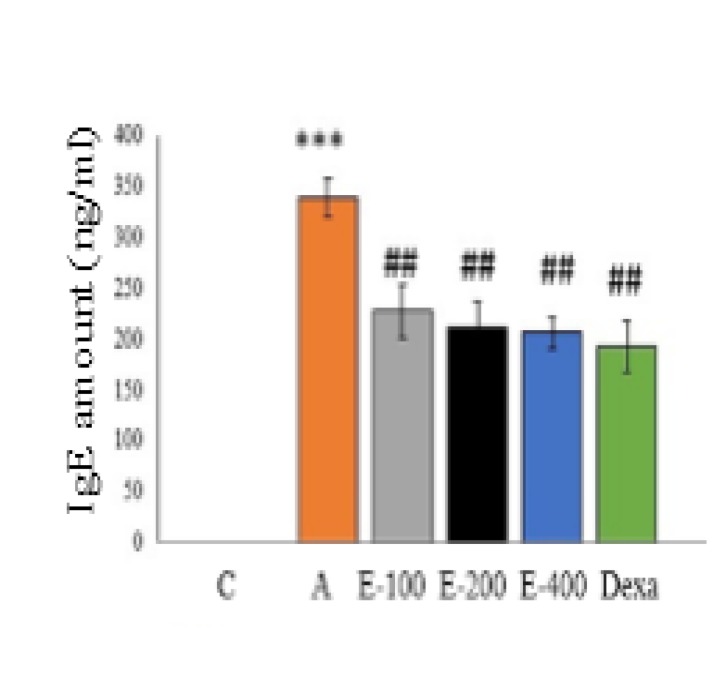
The effect of aqueous extract of *P. atalantica *gum on the amount of IgE antibody production in different groups. Asthmatic group compared to control group, ***p<0.001. Different treated groups compared to asthmatic group, ##p<0.01

IgE antibody production in asthmatic group was significantly increased in compared to control group (p<0.001), also its amount decreased significantly in mice treated with the aqueous extract of *P. atlantica *gum and dexamethasone compared to the asthmatic group (Figure 5) (p<0.01 for all cases), this effect was not dependent on the concentration of the extract. Various concentrations of the extract and dexamethasone did not show different significant effects in terms of IgE production level.

**Assessment of cytokines in culture supernatants**

**Figure 6:**
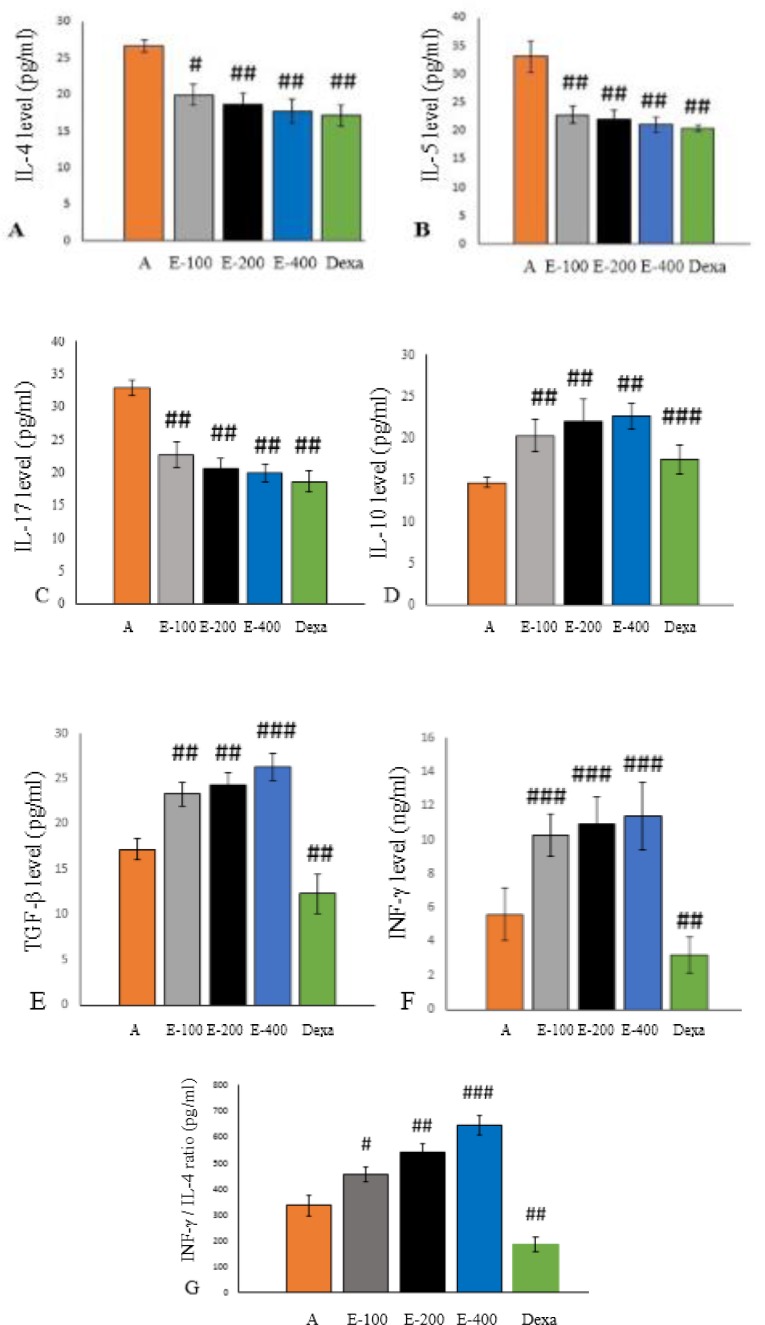
Effect of different concentrations of aqueous extract of *P. atalantica gum *and dexamethasone on the production of IL-4 (A), IL-5 (B), IL-17 (C), IL-10 (D), TGF-β (E), INF-γ (F) and IFN-γ/IL-4 ratio (G) by splenocytes of treated mice. #p<0.05, ##p<0.01, ###p<0.001, treated groups compared to asthmatic group

Analysis of splenocyte sample suspensions showed that the levels of IL-4 (Figure 6A), IL-5 (Figure 6B), and IL-17 (Figure 6C) cytokines were significantly higher in the asthmatic group compared to the all group. Furthermore, following oral administration of different concentrations of aqueous extract of *P. atlantica *gum and dexamethasone, levels of IL-4, IL-5, and IL-17 significantly decreased compared to the asthmatic group (p<0.05 to p<0.01). The levels of IL-10 (Figure 6D), TGF-β (Figure 6E), and INF-γ (Figure 6F) significantly increased in extract-treated groups compared to the asthmatic group (p<0.01 to p<0.001). However, levels of INF-γ (Figure 6F) and TGF-β (Figure 6E) were significantly decreased in dexamethasone-treated group compared to the asthmatic group (p<0.01). Dexamethasone showed weaker effect on the level of IL-10 cytokine than the three different concentrations of the extract.

**Evaluation of IFN-γ/IL-4 ratio**

The IFN-γ/IL-4 ratio was calculated after converting the unit (ng/ml) of interferon gamma (INF-γ) to pg/ml. The IFN-γ/IL-4 ratio in different concentrations of the extract significantly increased in a dose-dependent manner (for the extract) compared to asthmatic group (p<0.05 to p<0.001). The IFN-γ/IL-4 ratio in dexamethasone treated group was also significantly lower than in groups treated with different concentrations of the extract (p<0.01).

## Discussion

Medicinal plants play a key role in maintaining the health of individuals and communities. The medicinal value of these plants depends on their chemical composition, which have certain physiological effects on the human body system. The most important of these active agents are alkaloids, tannins, flavonoids, and phenolic compounds. Most herbs are rich sources of natural antioxidants that boost the antioxidant defense and reduce the incidence of certain diseases, such as cancer, heart diseases and stroke (Lü et al., 2010 ▶). *P. atlantica* gum as a rich source of phenolic compounds is used in traditional medicine as an antimicrobial, antifungal, anti-inflammatory, anti-viral, anti-rheumatic, and anti-cancer drugs (Haghdoost et al., 2013 ▶). The results of this study showed that oral administration of aqueous extract of *P. atlantica *gum had an anti-inflammatory effect against allergic asthma in mice. 

Administration of three concentrations (100, 200 and 400mg/kg) of aqueous extract of *P. atlantica *gum as well as dexamethasone reduced total number of cells, BALF eosinophils counts and OVA-specific IgE production. At 400mg/kg, the extract was more effective in decreasing the cell number and IgE level compared to the other concentrations; however, the effects of 400 mg/kg dose were not significantly different from dexamethasone.

Th1, Th2, Th17 and regulatory T cells cytokine levels were evaluated after administration of *P. atlantica *gum aqueous extract. Following induction of allergic asthma and administration of aqueous extract of *P. atlantica *gum, Th2 (IL-5 and IL-4) and Th17 cytokines (IL-17) significantly decreased, which did not vary significantly among different doses of the extract. Cytokines levels of both regulatory T cells (TGF-β and IL-10) and Th1 cells (INF-γ) significantly increased in treated groups. TGF-β and INF- γ levels dropped significantly in the group treated with dexamethasone compared to the asthmatic and treated groups with the various doses of extract. Recent studies indicated that dexamethasone non-selectively decreases and suppresses all immune responses (Assaf et al., 2017 ▶). The present study proved that dexamethasone decreases production of all these cytokines except IL-10. Minayan et al. (2015) ▶ studied the anti-inflammatory effects of *P. atlantica *gum extract in the inflammatory disease of acetic acid-induced colitis in rats. These researchers reported that prescribing gum extract reduced the amount of myeloperoxidase as an inflammatory marker and pathological damage to the intestinal tissue. They further analyzed the gum extract using GC/MS and reported that volatile oily compounds comprised 90% of the extract (41.23% α-pinene, 6.85% β-pinene, and 39.5% trans- verbenol) (Minaiyan et al., 2015 ▶). 

Various studies showed that IL-1, IL-6, and TNF-α play a key role in invoking inflammatory cells and tissue damage in inflammatory bowel diseases. In another study, the SHI-219 reduced the cellular and pathological symptoms of inflammatory disease, so it can be concluded that the use of this extract reduces the intensity of factors involved in inflammation and tissue damage (Murano et al., 2006 ▶). Also, it was shown that the levels of IL-6 and acute phase reactant proteins decrease significantly in Crown’s disease after treatment with *P. atlantica *gum extract (Kaliora et al., 2007 ▶). 

The polarization of immune responses towards Th2 cells that it has destructive effects on lung with the secretion of IL-4 and IL-5 cytokines indicating activation of this pathway during allergic asthma. IL-4 cytokine plays an important role in differentiating Th0 cells into Th2 cells and changing the immunoglobulin class to IgE. This cytokine also suppresses the differentiation of Th0 cells to Th1 (Bahrami et al., 2014 ▶). As our results indicated, an increase in interferon-gamma following oral administration of the aqueous extract of *P. atlantica *gum is indicative of a decrease in IL-4 and an increase in Th0 cell differentiation towards Th1 cells. 

Various studies showed that IL-5 cytokine plays an important role in the eosinophilia of the respiratory tract. This cytokine regulates eosinophilic growth, differentiation, maturity, binding, secretion and apoptosis (Rincon et al., 2012 ▶). Therefore, a reduction in this cytokine has a direct impact on the performance of eosinophils, as endorsed by the results of this study. 

Regulatory T cells are one the main producers of TGF-β and IL-10 cytokines, which regulate the immune response of Th2 cells and IgE production. The role of these cells in inhibiting the proliferation and function of other T lymphocytes was previously reported (Abedian kenari et al., 2009 ▶). Recent studies showed that regulatory T-cells in the lung of patients with allergic asthma, can overcome the responses of Th2/Th1. (Finotto et al., 2007 ▶). Our cytokine assay results revealed that the aqueous extract of *P. atlantica *gum significantly increased the production of regulatory cytokines. 

Inflammation plays a major role in asthma pathophysiology. Activation of various immune cells and the production of various mediators lead to bronchial inflammation and airway obstruction, as well as clinical manifestations such as coughing, wheezing and dyspnea (Hansbro et al., 2011 ▶). As mentioned above, Th17, Th2, Th1, Treg, neutrophils, macrophages, dendritic cells, epithelial cells, and smooth muscle cells generate cytokines associated with asthma disease. increased levels of such cytokines were seen in clinical specimens and asthmatic mice models (Esmaili Gourvarchin Galeh et al., 2018 ▶). Inflammatory cells contribute to the pathogenesis of asthma by secreting pro-inflammatory cytokines including TNF-α, IL-6, and IL-1. 

Th2 cytokines such as IL-4, IL-5, IL-13, and IL-9 also have the same effects on asthma. The use of 5-hydroxy methyl furfural with suppressive effects on IL-4 production, a key cytokine driving Th-2 immune responses, could effectively alleviate asthma (Khodaei et al., 2017 ▶). Research on the pathogenesis of autoimmune diseases showed significant decreases in Th1/Th2 (i.e. IFN-γ/IL-4) ratios in some diseases, (Tripathi et al., 2013 ▶). Therefore, any agent that modulates the Th1/Th2 balance through shifting from Th2 to Th1 pole can improve these diseases. A main finding of the present study was that treatment with the extract of *P. atlantica *gum increased the Th1/Th2 ratio and modulated the IFN-γ/IL-4 balance toward the Th1 cytokine.

The histopathological results of our study showed that all different concentrations of the aqueous extract of *P. atlantica* gum and dexamethasone reduced lung tissue damage. Also, 400mg/kg of the extract showed more intensive effects compared to dexamethasone. Considering the above-mentioned factors involved in asthmatic lung tissue damage, it is recommended to conduct a study on the molecular mechanisms involved in the tissue repair and reduction of inflammation by the aqueous extract of *P. atlantica *gum. 

Although symptoms of allergic inflammation of the respiratory tract and allergic asthma can be reduced and suppressed by some drugs, this disease cannot be cured completely. The results of our study signify that main effects of the plant extract are suppression of Th2 pathway and stimulation of regulatory T cells. The plant extract has a high potential for increasing the level of INF-γ (Th1 pathway), and IL-10 and TGF-β cytokines compared to dexamethasone as a general immunosuppressive agent. Therefore, aqueous extract of *P. atlantica* gum can be useful as a natural compound as a preventive drug for asthma and can be co-administered to reduce the required doses of synthetic drugs.
